# Effect of post-transplant glycemic control on long-term clinical outcomes in kidney transplant recipients with diabetic nephropathy: A multicenter cohort study in Korea

**DOI:** 10.1371/journal.pone.0195566

**Published:** 2018-04-18

**Authors:** Yong Chul Kim, Nara Shin, Sunhwa Lee, Huh Hyuk, Young Hoon Kim, Hyosang Kim, Su-Kil Park, Jang-Hee Cho, Chan-Duck Kim, Jongwon Ha, Dong-Wan Chae, Jung Pyo Lee, Yon Su Kim

**Affiliations:** 1 Department of Internal Medicine, Seoul National University Hospital, Seoul, Korea; 2 Clinical Medical Science, Seoul National University College of Medicine, Seoul, Korea; 3 Division of Kidney transplantation, Department of Surgery, Asan Medical Center and University of Ulsan College of Medicine, Seoul, Korea; 4 Department of Internal Medicine, Asan Medical Center and University of Ulsan College of Medicine, Seoul, Korea; 5 Department of Internal Medicine, Kyungpook National University School of Medicine, Daegu, Korea; 6 Department of Surgery, Seoul National University College of Medicine, Seoul, Korea; 7 Department of Internal Medicine, Seoul National University Bundang Hospital, Seongnam, Korea; 8 Department of Internal Medicine, Seoul National University Boramae Medical Center, Seoul, Korea; 9 Department of Medical Science, Seoul National University College of Medicine, Seoul, Korea; 10 Kidney Research Institute, Seoul National University College of Medicine, Seoul, Korea; Ospedale San Raffaele, ITALY

## Abstract

**Purpose:**

Diabetic nephropathy is the leading cause of end stage renal disease. The number of kidney transplantation (KT) due to diabetic nephropathy is increasing and there is debate on glycemic control after KT. In this study, we used a multi-center database to determine the relationship between post-transplant glycemic control and the outcomes of KT in patients with diabetic nephropathy.

**Methods:**

We conducted a retrospective chart review of kidney transplant recipients (KTRs) with diabetic nephropathy from three tertiary hospitals to analyze the association between post-transplant glycemic control and the clinical outcomes of graft failure, including patient death and biopsy-proven acute rejection (BPAR). We assessed time-averaged glucose level and hemoglobin A1c (HbA1c) for 36 months after KT.

**Results:**

Among 3,538 KTRs, a total of 476 patients received kidney transplantation because of diabetic nephropathy. Mean time-averaged glucose and HbA1c levels were 147 ± 46 mg/dl and 7.7 ± 1.5%, respectively. Patients with diabetic nephropathy had poor graft and patient survival rate compared with non-diabetic nephropathy. Among KTRs with diabetic nephropathy, the highest quartile of time-averaged glucose was related to poor graft outcomes and the 3^rd^ quartile of time-averaged HbA1c was associated with significantly better graft outcomes than the 1^st^, 2^nd^ or 4^th^ quartiles. There were no significant differences in the risk of BPAR across the 4 quartiles of glucose and HbA1c.

**Conclusions:**

Strict glycemic control before KT might not be related to successful outcomes but poor glycemic control after KT is associated with poor graft outcomes. There was no significant relationship between pre- or post-transplant glycemic control and BPAR.

## Introduction

Diabetic nephropathy is the leading cause of end stage renal disease (ESRD). In the United States Renal Data System (USRDS) 2013 annual report, diabetes was the most common cause of ESRD at nearly 50% of total incident dialysis [1]. According to the 2013 ESRD Registry in Korea, the incidence rate of diabetes in ESRD is 48.0%. There are three choices for renal replacement therapy (RRT): hemodialysis, peritoneal dialysis and kidney transplantation. Hemodialysis is the most common RRT modality, however, the rate of kidney transplantation is on the rise. Moreover, when compared to hemodialysis, kidney transplantation in patients with diabetic nephropathy (DN) is associated with better outcomes in terms of both mortality and cardiovascular complications such as coronary artery and peripheral vascular events [2,3]. In the United States, the prevalence of DN in kidney transplantation patients was 27.6% in 2002 and 28.9% in 2012; DN was the main cause of primary renal disease [4].

Poor glycemic control in diabetic patients without nephropathy is a well-known risk factor for cardiovascular [5] and all-cause mortality [6]. Also, compared to other causes of primary renal diseases, diabetic nephropathy is associated with poor outcomes in terms of cardiovascular complications and mortality in patients with ESRD [7]. Although successful renal transplantation decreases cardiovascular morbidity and mortality compared to chronic dialysis therapy, diabetes is still a risk factor for poor outcomes among kidney transplant recipients (KTRs) [8,9].

Pancreas and islet transplantation is important in restoring the glycemic control through conferring insulin independence for KTRs with type I diabetes. It is well known that successful pancreas and islet transplantation is associated with improvements not only in kidney function and kidney graft survival rates [10,11], but also in cardiovascular [12] and cerebrovascular function [13,14] among type I diabetic ESRD patients.

The American Society of Transplantation (ATC) published guidelines for the care of KTR in 2009. They recommended targeting HbA1c around 7.0–7.5% and avoiding HbA1c ≤ 6.0%, especially if hypoglycemic reactions are common in the patient [15]. In the general diabetic populations, it is recommended to target HbA1c < 7.0% and less stringent HbA1c targeting (<8%) is recommended in the advanced diabetic population with complications such as microvascular or macrovascular disease [16]. DN is an advanced microvascular complication; optimizing glycemic control is needed to slow the progression of nephropathy. But glycemic control in KTRs is still up for debate. In a randomized control trial (RCT) of glycemic control in a cohort of type I diabetic KTRs, the standard treatment group showed a more than twofold increase in mesangial matrix expansion (an indicator of diabetic nephropathy) compared with an optimized treatment control group. However, the optimized group showed a higher incidence of severe hypoglycemic episodes than the standard treatment group [17]. Recently, one study revealed that poor pre-transplant glycemic control is associated with decreased post-transplant survival [18]. In this study, pre-transplant time-averaged HbA1c ≥ 8% appeared to be associated with higher all-cause and cardiovascular mortality, but not with post-transplant graft outcomes or delayed graft failure. Moreover, this study showed no evidence to recommend intensive glycemic control after kidney transplantation. Wiesbauer et al. reported that maximum glucose levels but not HbA1c predicted survival in diabetic patients who underwent kidney transplants [19]. Ramirez et al. evaluated the association between preoperative and chronic glycemic control and clinical outcomes such as graft rejection, infection and hospital admission after kidney transplantation [20]. Their results showed that in the first 12 months after kidney transplantation, perioperative or chronic glycemic control was not associated with post-transplant outcomes. As such, it seems that near normal glycemic targets are not necessary for managing hyperglycemic after kidney transplantation; the effect of post-transplant glycemic control on long-term clinical outcomes was not clearly determined.

The objective of this study was to examine the association between post-transplant glycemic control and long-term clinical outcomes of transplantation (graft survival and graft rejection). We hypothesize that poor glycemic control after kidney transplantation is associated with poor graft outcome.

## Methods

### Patients

We performed a multicenter cohort study including patients admitted to three tertiary hospitals: Seoul National University Hospital (SNUH), Asan Medical Center University of Ulsan College of Medicine (AMC), and Kyungpook National University Hospital (KNUH). A total of 3,538 adult KTRs aged ≥18 years who underwent transplantation between 1997 and 2011 were included in this study. Patients who had multiple organ transplantation (liver, heart and especially pancreas/islet) were excluded. The patients with diabetes were selected regardless of the type (i.e., type I or II). Diabetes was diagnosed as follows (i.e., medical history of a 2-hour plasma glucose level ≥ 200 mg/dL during an oral glucose tolerance test or of a fasting glucose level ≥ 126 mg/dL; or HbA1c levels of at least 6.5%; or receiving treatment with oral hypoglycemic agents and/or insulin). A clinical diagnosis of DN included consistent urinary albumin-to-creatinine ratios ≥300 mg/g with no other causes of proteinuria. The present study was performed in accordance with the ethical standards of the Helsinki Declaration and was approved by the Institutional Review Boards and Research Ethics Committee of the three centers (SNUH, AMC and KNUH).

### Data collection

Patient characteristics were collected from a review of medical records. Transplant-related variables included age; gender; body mass index; primary cause of kidney failure; dialysis modality and duration; type of immunosuppressant; and history of pre-transplant hypertension, ischemic heart disease, and cerebrovascular disease. Pre-transplantation laboratory values for glucose, and HbA1c were obtained, and every 3months follow-up for glucose and HbA1c values were obtained. In addition, donor-related variables, including age and donor type were reviewed. Estimated glomerular filtration rate (eGFR) was calculated by the Modification of Diet in Renal Disease (MDRD) GFR equation [21].

### Immunosuppressive therapies

Induction therapy was mostly done with basiliximab or anti-thymocyte globulin (ATG) in high risk patients. Maintenance therapy was started with cyclosporine or tacrolimus, mycophenolate mofetil (MMF), and prednisolone.

### Outcomes

The primary endpoint was graft failure in transplant recipients. Graft failure was defined as composite of graft dysfunction that necessitated new renal replacement therapy after transplantation or patient death, which included death with functioning graft. The secondary outcome was a biopsy-proven acute rejection (BPAR) defined as a clinically meaningful acute rejection proven by kidney biopsy. Acute rejection episodes which were revealed in a protocol biopsy but not treated were not included.

### Statistical analysis

To investigate the effect of glycemic control on the outcomes, a comparison of outcomes among 4 quartiles of glucose and HbA1c was performed. Continuous variables were reported as means and standard deviations, and categorical variables were presented as frequencies with percentages. Continuous variables such as recipient and donor age and dialysis duration were compared using one-way ANOVA; categorical variables, such as proportion of comorbidities, cause of ESRD, and previous RRT modality, were compared using the Chi-square or Fisher exact test. The significance threshold for all analysis was set at p < 0.05. The independent risk factors for graft and patient survival were analyzed using multivariate Cox proportional hazard regression models. Appropriate covariates that were statistically significant in the univariate Cox proportional hazard regression analysis were included. All the variables were analyzed using the IBM SPSS software package (version 20.0; Armonk, NY, USA).

## Results

### Baseline patient characteristics

**Fig 1:**
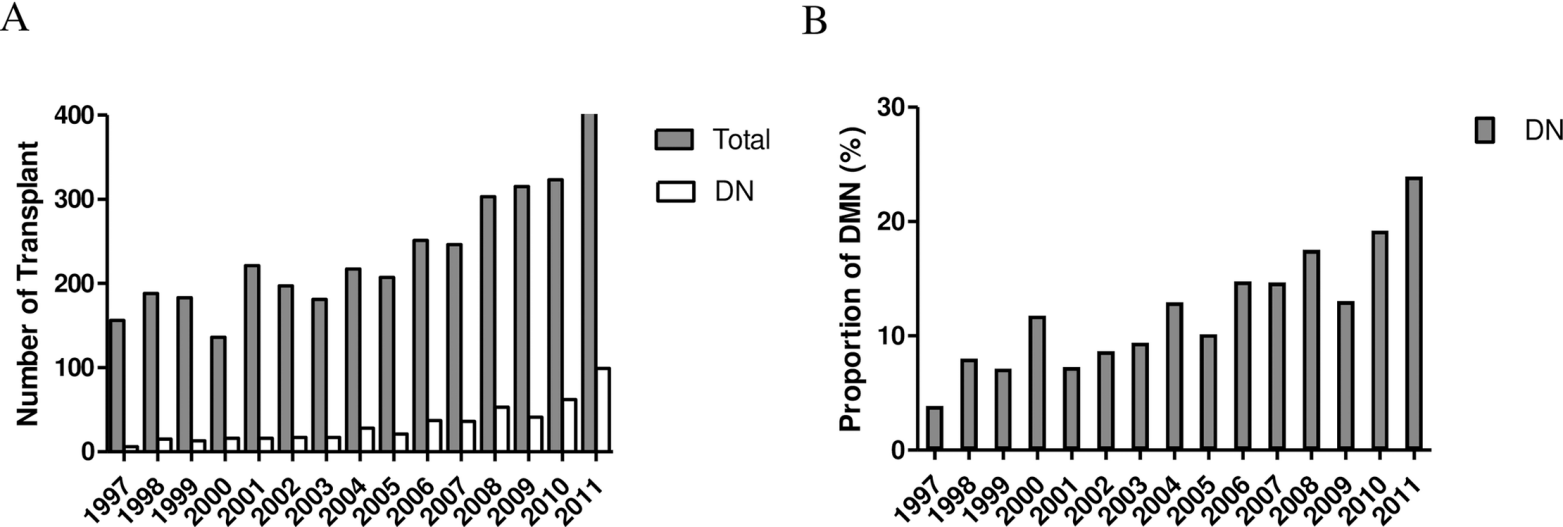
**Number (A) and proportion (B) of patients with diabetic nephropathy among total kidney transplantations from 1997 to 2011 in three hospitals (SNUH, AMC and KNUH).** DN, diabetic nephropathy; SNUH, Seoul National University Hospital; AMC, Asan Medical Center; KNUH; Kyungpook National University Hospital.

During the study period, 3,538 patients underwent kidney transplantation. The number of kidney transplants has increased each year and the proportion of kidney transplantation due to DN has also increased (Fig 1). Among 3,538 KTRs, a total of 476 patients received kidney transplantation because of diabetic nephropathy. Clinical, demographic and laboratory characteristics of patients are summarized in Table 1.

**Table 1:** Baseline characteristics by quartiles of time-averaged HbA1c levels.

	All patients	Quartile of time-averaged HbA1c				*P*
		Q1	Q2	Q3	Q4	
N	476	110	96	114	100	
Age (year, min-max)	50 ±10.2	47.1 ± 11.3	51.6 ± 9.2	50.8 ±10.0	50.0 ± 9.3	0.007
Gender (Male, %)	66.9	63.6	77.1	63.2	65.0	0.12
BMI (kg/m^2^)	23.4 ± 3.2	22.5 ± 2.8	24.0 ± 2.9	23.4 ± 3.0	23.8 ± 3.8	0.005
Co-morbidity (%)						
Hypertension	89.5	83.6	93.8	93.9	87.0	0.03
Ischemic heart disease	15.0	7.3	22.9	14.9	16.0	0.02
Cerebrovascular disease	5.0	2.7	6.3	7.9	3.0	0.23
Donor factors						
Donor age (y)	40.5 ± 13.9	39.7 ± 14.2	41.1 ± 14.2	41.3 ± 12.9	39.6 ± 13.2	0.72
Gender (Male, %)	55.9	59.3	60.8	54.5	49.4	0.46
Donor type (%)						0.003
Living related	43.3	33.7	42.6	54.0	41.8	
Living unrelated	32.3	30.8	27.7	31.0	39.8	
Deceased donor	24.4	35.6	29.8	15.0	18.4	
Duration of dialysis (months)	28.0	32.6	27.5	27.6	24.0	0.37
Predialysis modality (%)						0.87
Preemptive	14.0	12.5	12.5	14.9	16.0	
Hemodialysis	68.8	71.2	68.8	70.2	65.0	
Peritoneal dialysis	15.0	12.5	16.7	13.2	18.0	
Mixed (HD+PD)	2.2	3.8	2.1	1.8	1.0	
Immunosuppressant						
Calcineurin inhibitor (%)	99.5	100	100	100	97.6	0.07
Cyclosporine A	46.4	35.5	46.7	53.2	46.4	0.19
Tacrolimus	53.1	64.5	53.3	46.8	53.2	
Antimetabolite (%)	96.3	96.7	97.6	96.1	94.9	0.83
Baseline laboratory finding						
HbA1c (%)	7.5 ± 1.7	6.6 ± 1.4	6.9 ± 1.2	7.7 ± 1.3	8.8 ± 2.1	<0.001
Glucose (mg/dl)	194 ± 113	171 ± 98	180 ± 100	223 ± 131	197 ± 113	0.005
Albumin (g/dl)	3.4 ± 0.6	3.6 ± 0.6	3.4 ± 0.6	3.4 ± 0.6	3.4 ± 0.6	0.14
Hemoglobin (mg/dl)	10.7 ±1.5	10.7 ±1.5	10.6 ±1.8	10.5 ±1.6	10.3 ±1.9	0.37

Data are presented as medians (range) or frequencies (percentage). HbA1c, hemoglobin A1c; BMI, body mass index; HD, hemodialysis; PD, peritoneal dialysis

Data was collected for patients with DN from time of transplant to 36 months follow up. Of the 476 patients included in the data analysis, the majority were male (66.9%) and mean age at time of transplantation was 50 ± 10.2 years. In addition, 43.3% of patients received living-related transplants, 32.3% living-unrelated transplants, and 24.4% deceased-donor transplants. The mean HbA1c before transplantation was 7.5 ± 1.7% and the mean random glucose level was 194 ± 113 mg/dl.

Compared with non-DN, patients with DN were older and had higher BMI. Donors of DN group was older and there were more living unrelated donors. There was no significant difference in duration of dialysis, dialysis modality before transplantation, immunosuppressant use, and 1-year eGFR between the two groups. Baseline characteristics of the two groups did not fully match (Table 2).

**Table 2:** Baseline characteristics of non-DN versus DN.

	NON-DN	DN	*P*
N	3052	476	
Age (year, min-max)	40.7 ± 11.1	50.0 ±10.2	<0.001
Gender (Male, %)	58.1	66.9	<0.001
BMI (kg/m^2^)	22.2 ± 3.1	23.4 ± 3.2	<0.001
Co-morbidity (%)			
Hypertension	83.3	89.5	<0.001
Ischemic heart disease	2.4	15.0	<0.001
Cerebrovascular disease	2.2	5.0	<0.001
Donor factors			
Donor age (y)	39.0 ± 12.0	40.5 ± 13.9	0.007
Gender (Male, %)	57.4	55.9	0.17
Donor type (%)			0.001
Living related	53.4	43.3	
Living unrelated	22.8	32.3	
Deceased donor	23.8	24.4	
Duration of dialysis (months)	30.9 ± 38.4	28.0 ± 30.8	0.21
Cause of ESRD (%)			<0.001
DM	0	100	
CGN	24.8	0	
HTN	7.7	0	
others	67.5	0	
Predialysis modality (%)			0.67
Preemptive	11.7	14.0	
Hemodialysis	70.8	68.8	
Peritoneal dialysis	15.5	15.0	
Mixed (HD+PD)	2.0	2.2	
Immunosuppressant			
Calcineurin inhibitor (%)	100	99.5	0.11
Cyclosporine A	47.9	46.4	
Tacrolimus	52.1	53.1	
Baseline laboratory finding			
HbA1c (%)	5.8 ± 1.9	7.5 ± 1.7	<0.001
Glucose (mg/dl)	112 ± 41	194 ± 113	<0.001
Albumin (g/dl)	3.7 ± 0.5	3.4 ± 0.6	<0.001
Hemoglobin (mg/dl)	10.5 ± 1.8	10.7 ± 1.5	0.55
1-year Creatinine (mg/dl)	1.28 ± 0.67	1.30 ± 0.74	0.63
1-year eGFR	66.3 ± 19.5	63.9 ± 19.5	0.59

Data are presented as medians (range) or frequencies (percentage). DN, diabetic nephropathy; BMI, body mass index; DM, diabetes mellitus; CGN, chronic glomerulonephritis; HTN, hypertension; HD, hemodialysis; PD, peritoneal dialysis; eGFR, estimated glomerular filtration rate.

### Comparison of post-transplant outcomes between diabetic nephropathy and non-diabetic nephropathy

**Fig 2:**
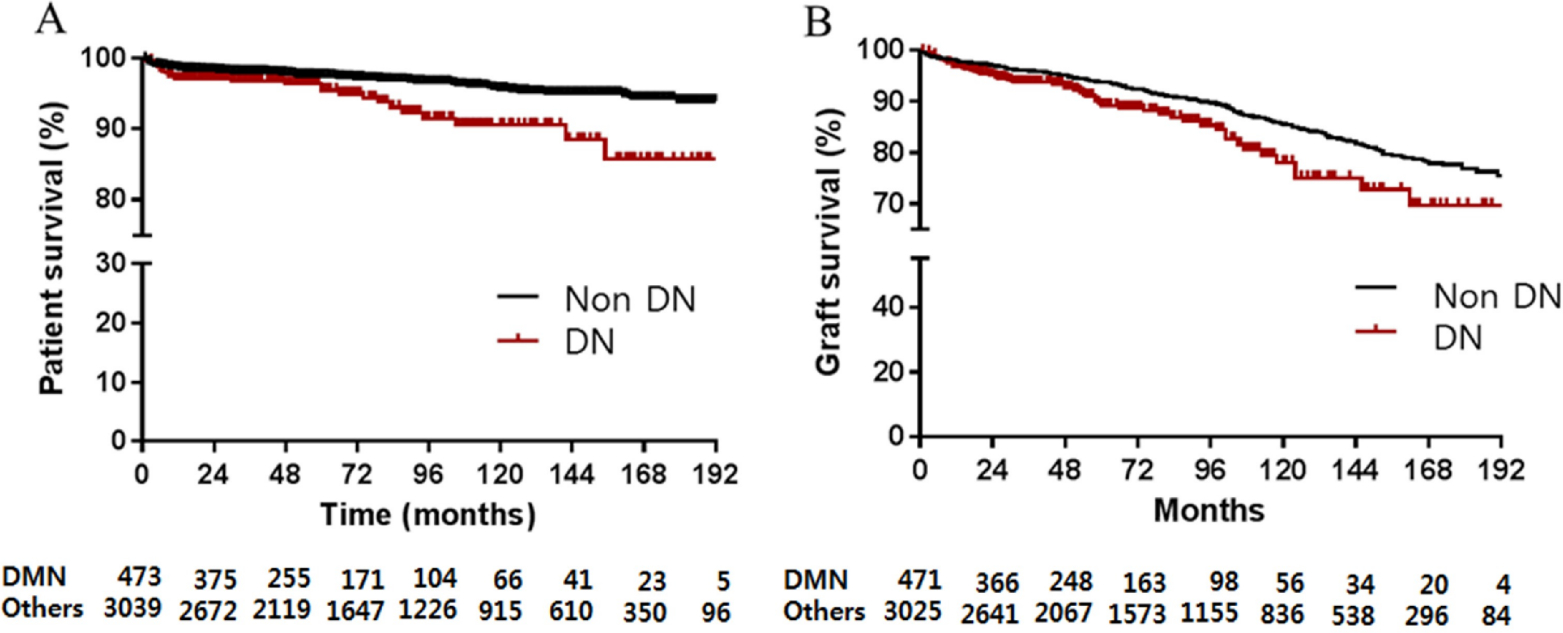
**Patient survival (A) and graft survival (B) for kidney transplant patients.** DN, diabetic nephropathy.

During the follow-up period, 9.3% (284/3052) of non-diabetic patients developed post-transplant DM. Sixty graft failures (12.6%) and 30 deaths (6.3%) occurred in patients with DN, compared to 354 graft failures (11.6%) and 117 deaths (3.8%) in patients with non-DN. Post-transplant patient survival of KTRs with DN was poorer than that of KTRs with non-DN (*p* <0.001; Fig 2A). The survival rate of DN and non-DN was 97.0% and 98.5% at 1 year follow up, and 95.4% and 97.5% at 5 years. In addition, graft survival of KTRs with DN was inferior to graft survival of non-DN (*p* <0.001; Fig 2B). The graft survival rate of DN versus non-DN was 96.8% and 98.0% respectively at 1 year, and 89.2%, respectively, and 93.8% at 5 years. Death-censored graft survival rate was also decreased in KTRs with DN (*p* = 0.035). There was no difference in 1-year serum creatinine (DN: 1.30±0.80 vs non-DN: 1.28±0.73 mg/dl, *p* = 0.628), and 1-year estimated GFR in patients with diabetic nephropathy (DN: 65.6±21.2 vs non-DN: 66.1±19.1, *p* = 0.588).

### Post-transplant glycemic control and risks of graft failure

**Fig 3:**
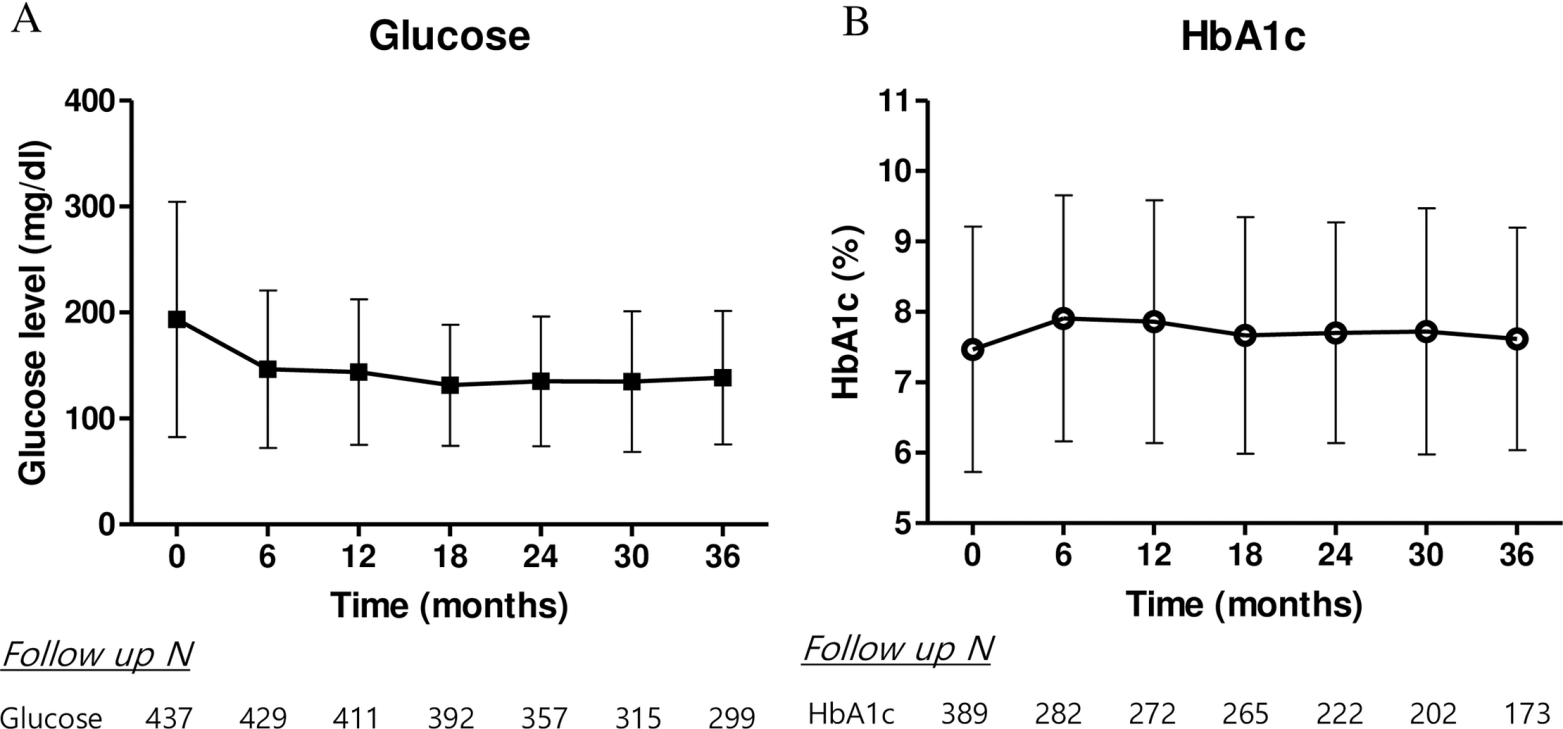
Transition of post-transplant glycemic control by serum glucose level and HbA1c.

The median follow up duration for patients with diabetic nephropathy was 49.9 months. During the follow up period, events of graft failure were confirmed in 62 (13%) patients in diabetic nephropathy. The changes in fasting glucose levels and HbA1c every 6 months were shown in Fig 3. Each post-transplant HbA1c was higher than baseline but within the range of 7–8% (baseline HbA1c = 7.5±1.7 vs. time-averaged HbA1c = 7.7±1.5, *p* < 0.001). Post-transplant glucose levels were lower than baseline levels, in the range of 120–160. The mean time-averaged glucose levels and HbA1c at 36 months were 147 ± 46 mg/dl and 7.7 ± 1.5%, respectively.

**Fig 4:**
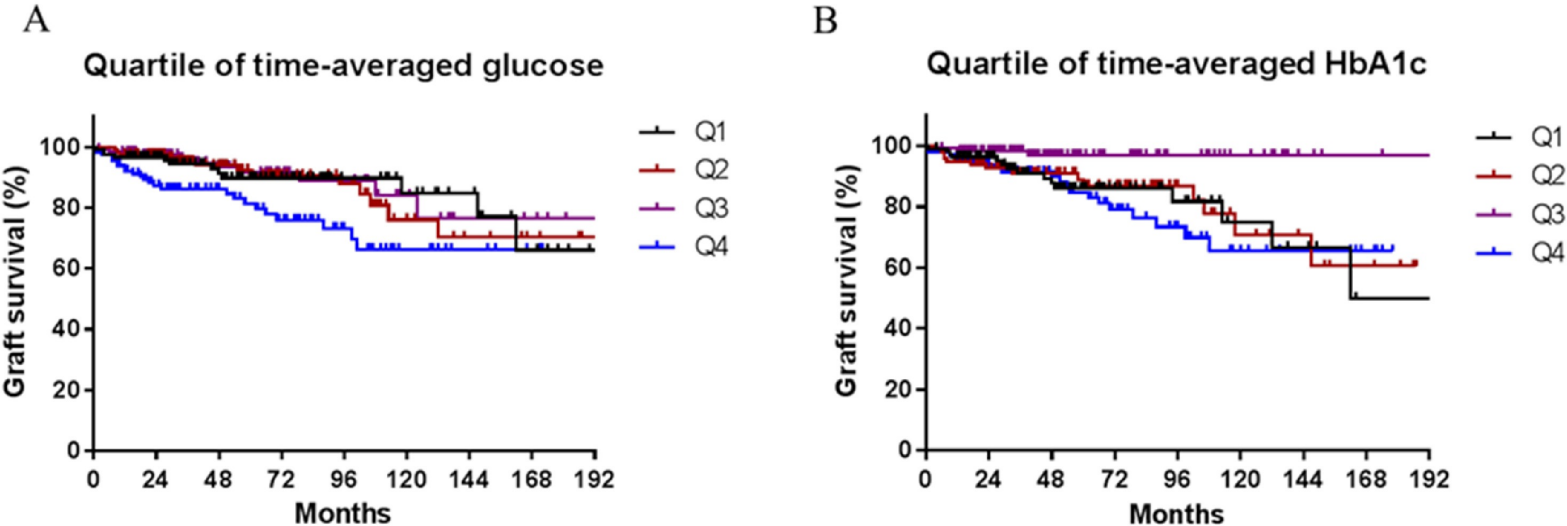
Kaplan-Meier estimates according to quartiles of glucose and HbA1c. Graft survival included graft failure and patient death with functioning graft.

The highest quartile of time-averaged glucose level predicted poor graft survival in the unadjusted model (*p* = 0.01; Fig 4A). In addition, the 3^rd^ quartile of time-averaged HbA1c showed good graft survival compared to the other quartiles (*p* = 0.006; Fig 4B).

**Fig 5:**
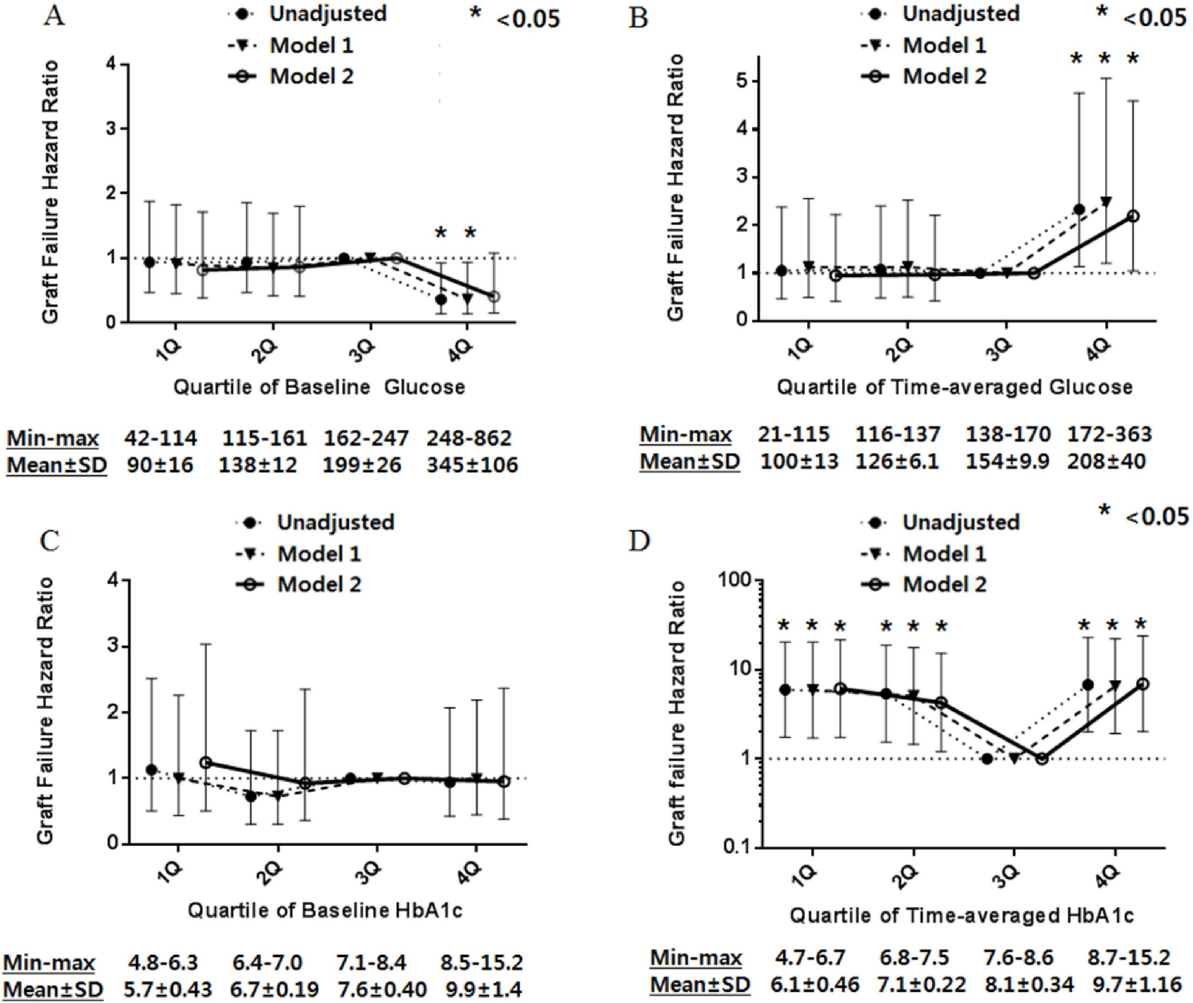
Hazard ratios of graft failure by serum glucose using standard Cox proportional hazards regression (A) and a time-averaged model (B). Hazard ratios of graft failure by HbA1c using standard Cox proportional hazards regression (C) and a time-averaged model (D). Model 1 is adjusted for age and gender. Model 2 is adjusted for age, gender, comorbidities (hypertension, ischemic heart disease), donor age, donor type, baseline hemoglobin level, and BPAR.

Next, we performed a Cox regression analysis. Fig 5 shows the unadjusted and adjusted graft failure hazard ratios (HRs) for the quartile groups based on baseline glucose, baseline HbA1c, time-averaged glucose, and time-averaged HbA1c. In the unadjusted model and in the model adjusted only for age and gender, the highest quartile of baseline glucose showed low HR for graft failure, but in the model adjusted for age, gender, comorbidities, age of donor, donor type, and BPAR, there was no significant association (Fig 5A). Using time-averaged glucose level as a modifier, highest quartile of time-averaged glucose showed high HR for graft failure in unadjusted model, the model adjusting for age and gender, and the model adjusting for age, gender, comorbidities, age of donor, donor type and BPAR (Fig 5B).

HbA1c, an index of glycemic control, was used for analyze the effect of post-transplant glycemic control on graft failure. In Cox regression analysis, baseline HbA1c was not significantly associated with graft failure (Fig 5C). However, in the analysis using time-averaged HbA1c quartiles, the 1^st^ (HR 6.46, 95% CI 1.82–22.9, *p* = 0.004), 2^nd^ (HR 4.61, 95% CI 1.29–16.38, *p* = 0.02) and 4^th^ quartiles (HR 7.89, 95% CI 2.28–27.30, *p* = 0.001) were related to poor graft outcomes compared with the 3^rd^ quartile (7.6–8.6%), after adjusting age, gender, comorbidities, donor age, donor type, and BPAR (Fig 5D).

### Post-transplant glycemic control and risk of BPAR

**Fig 6:**
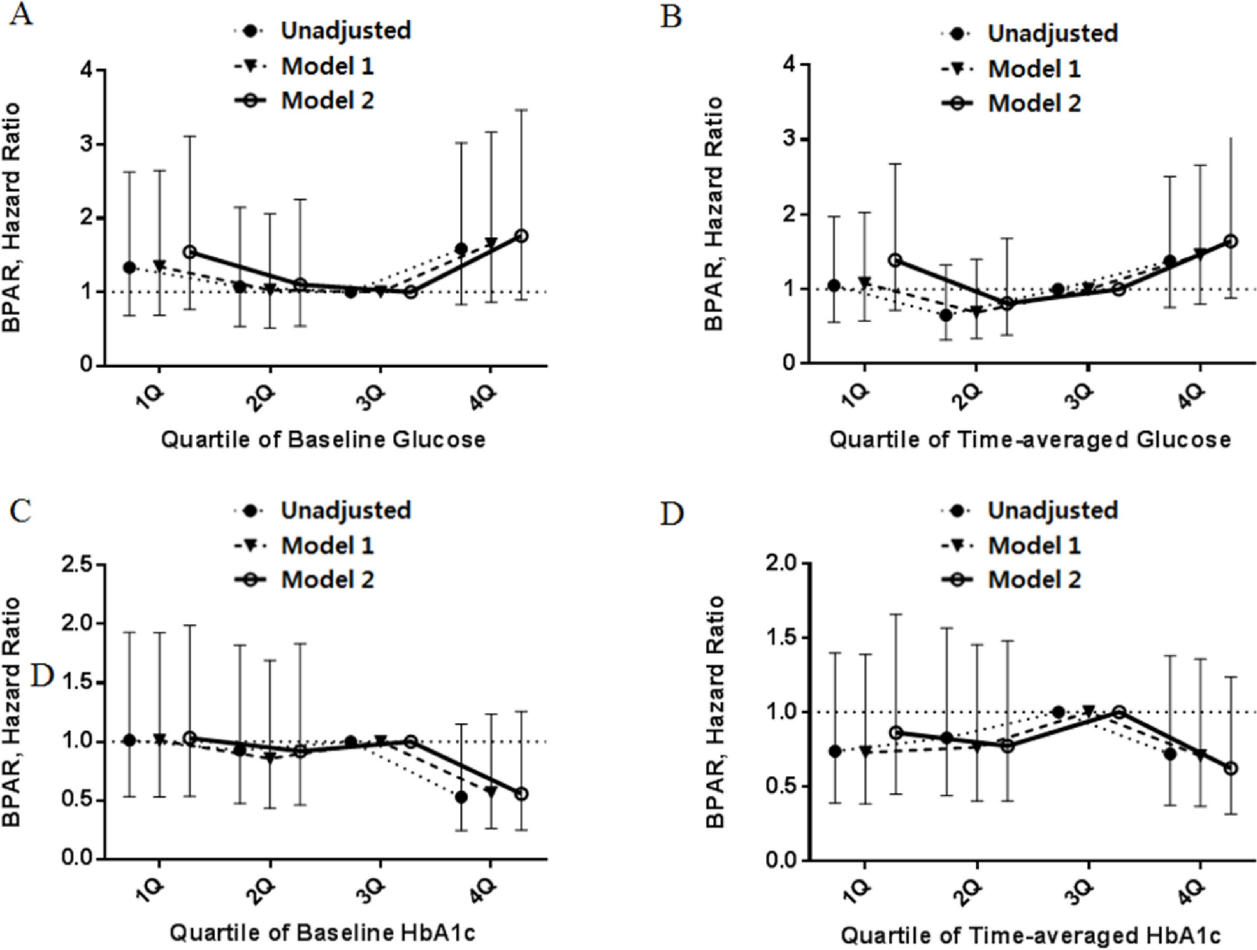
Hazard ratios of BPAR by serum glucose using standard Cox proportional hazards regression (A) and a time-averaged model (B). Hazard ratios of BPAR by HbA1c using standard Cox proportional hazards regression (C) and a time-averaged model (D). Model 1 is adjusted for age and gender. Model 2 is adjusted for age, gender, comorbidities (hypertension, ischemic heart disease), donor age, and donor type.

During the follow up period, episodes of BPAR were confirmed in 81 patients (17.0%) with diabetic nephropathy. There was no significant relationship between BPAR and baseline/time-averaged glucose or between BPAR and HbA1c levels (Fig 6).

## Discussion

This multicenter retrospective cohort study reports the clinical outcomes of kidney transplantation in diabetic nephropathy and its relationship with post-transplant glycemic control. Graft and medical outcomes after kidney transplantation for diabetic nephropathy were poor compared to outcomes for patients with non-diabetic nephropathy. In addition, post-transplant glycemic control, assessed by time-averaged glucose levels and HbA1c, affected graft survival. The HbA1c group with 7.6–8.6% showed the best graft outcome. However, pre-transplant glycemic control was not associated with graft survival. Our results suggest that post-transplant glycemic control is more important than pre-transplant glycemic control for long-term graft outcomes. Acute rejection was not associated with pre- or post-transplant glycemic control.

The relationship between post-transplant glycemic control and clinical outcomes after kidney transplantation in clinical studies is controversial. Hyperglycemia is associated with ischemic reperfusion injury in animal models [22]. Also, in human kidney transplantation, hyperglycemia reportedly increases ischemic injury [23] and mesangial matrix expansion [17]. Wiesbauer et al. reported that maximal glucose levels were associated with mortality [19]. Hermayer et al. conducted a small, single-center, RCT with 93 patients who underwent kidney transplantation, randomized to either the intensive group with intravenous insulin or the standard treatment group with subcutaneous insulin. Results suggested that the intensive glycemic control after kidney transplant increased risk for rejection episodes, although delayed graft function, which was the primary outcome, was not statistically different. In this trial, the target of intensive glycemic control was relatively strict (blood glucose 70–110 mg/dl), so that hypoglycemic event was increased in this group. It is rational to assume that it might be related with the increased event of rejection. But, the authors reported that none of patients with graft rejection had hypoglycemic event in the intensive group [24]. Recent study showed early hyperglycemia after kidney transplantation was associated with increased risk of post-transplant diabetes, and patients with new onset diabetes after transplantation (NODAT) had higher rates of graft loss. Also, in accordance with our study, elevated blood glucose level and HbA1c at 3 months after transplantation was related with graft failure [25].

Glycemic control in kidney transplantation is challenging. The main pathophysiological mechanism of hyperglycemia after transplantation is pancreatic beta cell dysfunction in the context of insulin resistance. There are immunosuppressive agents which causes dysglycemia: corticosteroids, calcineurin inhibitors including tacrolimus and cyclosporine, as well as the mammalian target of rapamycin inhibitors (sirolimus and everolimus). In particular diabetic nephropathy patients who underwent kidney transplantation had difficulty controlling their diabetes because of complications, such as autonomic neuropathy. Therefore, the American Society of Transplantation (ATC) recommends targeting HbA1c 7.0–7.5% and avoiding targeting HbA1c ≤ 6.0% [15].

In our study, strict glycemic control as well as poor glycemic control were related to poor graft outcomes, which supports the ATC recommendations for glycemic control, although the exact range of HbA1c does not fit to that. We suggest that HbA1c might be more important parameter than glucose to survey for post-transplant glycemic control because, unlike glucose, it seems to be associated with graft outcome.

Our study has some limitations. First, as with all retrospective studies, our data cannot be interpreted causally. Second, the data for glucose levels could contain both fasting and random glucose levels because we cannot recognize whether the blood samples were collected before or after a meal. Third, we classified the laboratory findings into quartiles using cutoffs suggested by the data, rather than by the clinical literature. Furthermore, we had no information regarding diabetes medications, and whether patients were taking oral agents or insulin. This may be a confound as Wiesbauer et al. suggested that diet and oral medications seem to be superior to subcutaneous insulin obtaining optimal glycemic control [19]. Also the number of patient deaths and graft failures was small, which may have reduced the power in our analyses.

However, to our knowledge, this study represents the largest cohort study of Asian kidney transplantation to date, using multicenter cohort data. Furthermore, we used both glucose levels and HbA1c as indices of glycemic control. By measuring time-averaged glucose and HbA1c, we were able to reduce observed variability over time and examine overall trends in the association between glycemic control and survival. However, these methods may mask significant changes in laboratory parameters that are important to survival.

In conclusion, our study suggests that strict glycemic control might not be necessary for managing hyperglycemia after kidney transplantation, and that a good glycometabolic control may improve particularly long-term graft outcomes. As a parameter of glycemic control after kidney transplantation, HbA1c may be superior to glycemia because it may predict graft outcomes.

## Supporting information

S1 File Individual patient information. (XLSX)
